# Association analysis of SNPs present in plasma with adverse events and population pharmacokinetics in Chinese sunitinib treated patients with renal cell carcinoma

**DOI:** 10.18632/oncotarget.23881

**Published:** 2018-01-03

**Authors:** Yuanyuan Zhang, Haixing Mai, Gang Guo, Guofang Bi, Guangtao Hao, Yuanyuan Li, Xiaofang Wang, Longmei Cheng, Jing Wang, Ruihua Dong, Zeyuan Liu, Lijun Chen, Hengyan Qu

**Affiliations:** ^1^ Department of Clinical Pharmacology, Academy of Military Medical Sciences Affiliated Hospital, 307 Clinical College, Anhui Medical University, Beijing 100071, China; ^2^ Department of Urology Department, Academy of Military Medical Sciences Affiliated Hospital, Beijing 100071, China; ^3^ Department of Urology Department, The General Hospital of the People's Liberation Army, Beijing 100853, China

**Keywords:** single-nucleotide polymorphisms, cell-free DNA, pharmacokinetics, sunitinib, renal-cell carcinoma

## Abstract

**Background:**

Sunitinib is a tyrosine kinase inhibitor with effective therapeutic outcomes in patients with renal-cell carcinoma. The study were to analyze the association of single-nucleotide polymorphisms present in cell-free DNA and pharmacokinetics with sunitinib treatment-emergent adverse events in Chinese patients with renal-cell carcinoma.

**Materials and Methods:**

We genotyped 8 keys SNPs in 6 candidate genes. The plasma concentrations of sunitinib and N-desethyl sunitinib were measured using a high performance liquid chromatography-tandam mass spectrometry method. Correlations between the single-nucleotide polymorphisms and adverse events were investigated by univariate and multivariate logistic regression and we quantitatively evaluated the effect of single-nucleotide polymorphisms on the pharmacokinetics of sunitinib by using a population PK model.

**Results:**

Necessary dose reductions of sunitinib were significantly correlated with SNP rs1933437 in *FLT3*. A higher severity of AEs were collected with SNP rs2032582 in *ABCB1* and rs1800812 in *PDGFRα*. Thrombocytopenia was collected with rs1800812 in *PDGFRα*. Our study provides a population PK model of sunitinib with the *ABCB1* genotype as a predictive covariate for apparent oral clearance.

**Conclusions:**

Our study preliminarily confirmed the hypothesis that the pharmacokinetics of sunitinib is affected by the SNPs of enzyme in Chinese renal-cell carcinoma patients, and this affects the different distribution and severity of adverse events of sunitinib.

## INTRODUCTION

Sunitinib, a small-molecule receptor tyrosine kinase inhibitor (TKI), has been approved as the first- or second-line treatment for patients with metastatic renal cell carcinoma (mRCC) [1–3]. The recommended dose and schedule for sunitinib is 50 mg each day given orally for 4 consecutive weeks followed by 2 weeks-off per treatment cycle (schedule 4/2). According to the pharmacokinetic studies, Sunitinib is converted to its active metabolite N-dehydro-sunitinib (SU12662). What's more, SU12662 has a similar inhibitory profile to sunitinib [4, 5]. And the total active drug in plasma should be combined by sunitinib plus SU12662. Sunitinib has demonstrated favorable clinical benefits in comparison with interferon therapy [6, 7], such as better radiological response and survival.

Sunitinib's treatment-emergent AEs were hematological toxicities, such as thrombocytopenia, neutropenia, and anemia, and non-hematological toxicities such as hand-foot syndrome (HFS), hypertension, diarrhea, fatigue, and oral mucositis [8–11]. Compared with non-Asians, Asians are more likely to develop sunitinib-induced AEs and they are also more likely to have more severe AEs [6, 12]. Thus, sunitinib treatment-emergent AEs is a serious problem that should not be ignored, especially in Asian patients.

There is emerging evidences showing that variability in sunitinib-induced toxicity between different ethnic groups may be associated with SNPs in genes related to the pharmacokinetic pathways of sunitinib in patients with RCC [13–17]. Exploratory analyses have reported the targeted candidate genes including vascular endothelial growth factor receptors (*VEGFRs*) 1, 2, and 3; platelet-derived growth factor receptor (*PDGFR*) α and β; Fms-like tyrosine kinase 3 receptor (*FLT3*); the receptor encoded by the ret proto-oncogene (*RET*); and the pharmacokinetic related genes including cytochrome P450 1A1 (*CYP1A1*), cytochrome P450 3A5 (*CYP3A5*), ATP binding cassette member G2 (*ABCG2*) and ATP binding cassette member B1 (*ABCB1*) [14, 17, 18]. However, these studies were mostly conducted in Caucasian populations in North America or Europe and very little Chinese data has been reported [19, 20].

Studying the polymorphisms in these genotypes can be helpful to maximize the clinical benefits of sunitinib and optimize the therapeutic management strategy for Chinese RCC patients. According to a pharmacodynamic and pharmacokinetic meta-analysis [21], higher sunitinib exposure is associated with longer time to progression, longer overall survival (OS), greater reduction in tumor size, and increased risk of adverse events such as fatigue, hypertension, and neutropenia. Moreover, a retrospective analysis of 521 patients with mRCC treated with sunitinib in phase II and III trials showed that those who had a dose reduction because of toxicity had a similar day 29 trough level for sunitinib and its metabolites compared with those without a dose reduction [22]. The objective of the present study was to analyze the correlation of selected SNPs present in cell-free DNA with AEs and pharmacokinetics (PK) in Chinese RCC patients by using multivariate analyses and a well estimated population PK model. The primary goal was to generate information that can be used to optimize the therapeutic management strategy for Chinese RCC patients.

## RESULTS

### Baseline characteristic of Chinese patients with RCC

We collected 127 blood samples from 53 RCC patients who started sunitinib-treated between March, 2014, and January, 2016. Of 53 sunitinib-treated patients with RCC, 40 (75%) were males and 13 (25%) were females (Table 1). The median age was 54 years (range: 19-71). In all, 40 sunitinib-treated patients underwent renal surgery, and 20 were observed dose reductions within 1-4 cycles.

**Table 1:** Characteristics of RCC patients treated with sunitinib

Characteristic	Value (quartiles:25th and 75th percentile)	%
gender		
male	40	75.47
female	13	24.53
age at start sunitinib, yr	51.65 (45, 61)	
BSA	1.86 (1.74, 1.94)	
Prior nephrectomy		
yes	40	75.47
no	13	24.53
unknown		
ECOG performance status		
0	37	69.81
1	16	30.19
Ethnicity		
Chinese	53	100.00
No. of metastatic sites		
1	24	45.28
2	8	15.09
≥ 3	11	20.75
metastatic sites		
lung	19	35.85
liver	1	1.89
bone	4	7.55
lymph nodes	11	20.75
brain	1	1.89
kidney	11	20.75
Sunitinib, daily dose, mg, in first 4 cycles		
50 mg	34	64.15
37.5 mg	8	15.09
25 mg	11	20.75
dose reduction after cycle 1, 2 or 3		
yes		
men	14	26.42
women	5	9.43
total	19	35.85
no		
men	26	49.01
women	8	15.09
total	34	64.15

### Genetic polymorphisms analysis of Chinese patients with RCC

For each of these 8 polymorphisms, the respective genotypes and allele frequencies are given in Table 2. The SNPs were all in Hard-Weinberg equilibrium (*p* > 0.05). The allele frequencies of the genotyped polymorphisms were similar to those previously described elsewhere for Han Chinese in Beijing in a dbSNP database, except for SNPs rs2032582 and rs2231142. Their observed minor allele frequencies in the dbSNP database were slightly lower compared with our frequency of reportedfor rs2231142. In the case of rs2032582, there were only G and T alleles reported in the dbSNP database. However, in our study there were G, T and A alleles. The difference may because the patients that we collected were not all Han.

**Table 2:** Genotypes and allele frequencies of selected SNPs

Gene	RS ID	SNP	No. of patients	Genotype frequency	Allelic frequency	H-W equilibrium analysis	
						χ^2^	*p*
CYP3A4	rs35599367					N/A	N/A
		CC	53	100%	CC = 1		
		CT					
		TT					
CYP3A5	rs776746					0.78	0.38
		AA	2	3.77%	A = 0.25		
		AG	22	41.51%			
		GG	29	54.72%	G = 0.75		
ABCB1	rs2032582					0.31	0.86
		GG	10	18.87%	G = 0.43		
		GT/A	26	49.06%	T = 0.44		
		AA/TT/TA	17	32.08%	A = 0.12		
ABCG2	rs2231142					0.24	0.62
		AA	10	18.87%	A = 0.42		
		AC	24	45.28%			
		CC	19	35.85%	C = 0.58		
ABCB1	rs1128503					0.44	0.50
		CC	3	5.66%	C = 0.27		
		CT	23	43.40%			
		TT	27	50.94%	T = 0.73		
ABCB1	rs1045642					0.44	0.51
		CC	13	24.53%	C = 0.47		
		CT	24	45.28%			
		TT	16	30.19%	T = 0.53		
PDGFRA	rs1800812					0.36	0.55
		GG	34	64.15%	G = 0.74		
		TG	16	30.19%			
		TT	3	5.66%	T = 0.26		
FLT3	rs1933437					3.48	0.07
		CC	7	13.21%	C = 0.28		
		CT	16	30.19%			
		TT	30	56.60%	T = 0.72		

### Sunitinib treatment-emergent adverse events in RCC patients

Treatment-emergent adverse events (AEs) for patients who received sunitinib on schedule 4/2 were recorded. The when the analysis was limited to patients in first-line treatment only. The incidences of sunitinib treatment-emergent AEs by highest grade/severity are summarized in Table 3. These data show that 40 patients (75%) had grade 2 or grade 3 AEs, including HFS (42%), hypertension (34%), fatigue (32%), and thrombocytopenia (15%). Grade 1 or 2 AEs were a loss of appetite (35%), fatigue (32%), diarrhea (30%), hand-foot syndrome (HFS) (28%), thrombocytopenia (28%), leukopenia (24%) and hypertension (18%). No patients had grade 4 AEs. In total, 20 patients (38%) had a dose reduction due to toxicity during sunitinib treatment.

**Table 3:** Sunitinib treatment-emergent adverse events

Adverse events	Toxicity grade	No. of patients	% of patients
Thrombocytopenia	None	28	53
	1	15	28
	2	4	8
	3	4	8
Leukopenia	None	36	68
	1	13	25
	2	0	0
	3	2	4
Hypothyroidism	None	35	66
	1	3	6
	2	3	6
	3	1	2
Diarrhea	None	27	51
	1	16	30
	2	7	13
	3	1	2
Fatigue	None	17	32
	1	17	32
	2	16	30
	3	1	2
A loss of appetite	None	31	58
	1	19	36
	2	1	2
	3	0	0
Hand-foot syndrome	None	13	25
	1	15	28
	2	17	32
	3	5	9
Hypertension	None	23	43
	1	10	19
	2	7	13
	3	11	21

### Dose reduction, total trough level (TTL) and AEs study of sunitinib in RCC patients

**Figure 1:**
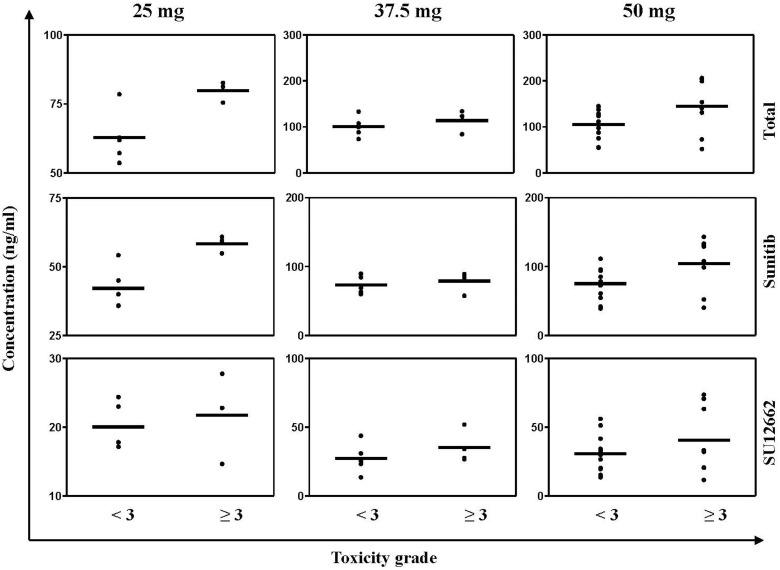
Steady state plasma concentration (Css) of sunitinib, SU12662 and (sunitinib + SU12662) of all patients who were treated by sunitinib The black bars represent the median Css.

We enrolled and analyzed data from 40 sunitinib-treated patients with RCC who had reached a steady state concentration. A steady-state concentration is considered representative of the entire measurement period, measured after 14 days of sunitinib treatment was used in our study. Among the 40 sunitinib-treated patients, 18 (45%) had a dose reduction because of sunitinib treatment-emergent severity AEs, primarily hematological. Therefore, 22 patients were consistently administered sunitinib at a dose of 50 mg per day. The median TTL among these patients who had grade ≥ 3 AEs was 140.81 (51.80–206.30) compared with 109.24 (54.31–199.74) ng/mL of the patients who had AEs of grade < 3 (*p* = 0.01). In 10 sunitinib-treated patients who had severe toxicity, the sunitinib administration dose was reduced to 37.5 mg per day. In these patients, the median TTL among those with grade ≥ 3 AEs was 114.49 (84.23–123.29) vs 107.60 (73.67–254.55) ng/mL among the other patients (*p* = 0.03). Moreover, 8 patients had a dose reduction to 25 mg per day and among these the median TTL of the patients who had grade ≥ 3 AEs was 77.02 (53.60–82.62) vs 61.90 (57.17–62.82) ng/mL among the other patients (Figure 1 and Supplementary Table 1). *t* test results of the two independent samples of various doses are shown in Table 4. And a general classification of samples in terms of whether the sample had reached the steady state concentration are shown in Supplementary Table 4.

**Table 4:** *T* test result of the two independent samples of various doses

Con. (ng/mL)	Toxicity grade	25 mg		37.5 mg		50 mg	
		mean ± SD	*p*	mean ± SD	*p*	mean ± SD	*p*
TTL	< 3	62.81 ± 9.56	0.45	100.76 ± 19.91	0.03^*^	105.53 ± 30.80	0.06
	≥ 3	80.11 ± 4.00		114.00 ± 21.39		145.10 ± 59.11	
Sunitinib	< 3	42.20 ± 7.67	0.18	72.78 ± 11.70	0.09	75.22 ± 21.56	0.07
	≥ 3	58.37 ± 3.13		78.91 ± 14.56		104.45 ± 38.77	
SU12662	< 3	20.02 ± 3.39	0.26	27.99 ± 10.05	0.08	30.70 ± 12.93	0.01^**^
	≥ 3	21.73 ± 6.62		35.09 ± 11.70		40.65 ± 24.71	

^*^p < 0.05 means statistically difference, ^**^p < 0.01 means statistically significant difference.

### Association between SNPs and AEs

Next, we identified SNPs associated with sunitinib-related toxicity. The univariate and multivariate logistic regression analyses for correlations between each of the genotyped SNPs and toxicity are listed in Table 5. In the multivariate analysis, SNP rs2032582 in *ABCB1* 2677 TT, AT or GT genotypes and rs1800812 in *PDGFRα* GG genotype were significantly correlated with grade 2 and grade 3 HFS (odds ratio [OR] 6.6, 95% confidence interval [CI] 1.2–37, *p* = 0.03; OR 6.6, 95% CI 1.4–31.4, *p* = 0.02; respectively). SNP rs1800812 in *PDGFRα* GG carriers was significantly more frequent in patients with thrombocytopenia (OR 5.2, 95% CI 1.3–21.8, *p* = 0.02). SNP rs776746 in *CYP3A5* GG were less likely to experience hypertension when compared with the AA or AG carriers (OR 0.3, 95% CI 0.1–0.9, *p* = 0.05). It is noteworthy that the *FLT* 738 TT carriers required fewer dose reductions (OR: 0.2; 95% CI, 0.1–0.9, *p* = 0.04).

**Table 5:** Univariate and multivariate analyses: association between SNPs and toxicity

	Group		Prevalence	Univariate		Multivariate	
				OR (95% Cl)	*P*	OR (95% Cl)	*P*
HFS	Gender	male vs	15/40	1			
		female	8/13	2.7 (0.7-9.7)	0.12		
	CYP3A5	AA+AG	10/24	1		1	
		GG	12/29	1.0 (0.3-3.0)	0.60	1.0 (0.3-4.0)	0.97
	ABCB1	other	5/16	1		1	
		TT+AT+GT	18/37	2.1 (0.6-7.2)	0.19	6.6 (1.2-37.0)	0.03
	ABCG2	CC+AC	18/43	1		1	
		AA	5/10	1.4 (0.4-5.5)	0.45	1.4 (0.2-7.9)	0.74
	ABCB1’	CC+CT	10/26	1		1	
		TT	13/27	1.5 (0.5-4.4)	0.33	0.9 (0.2-3.9)	0.90
	ABCB1’’	CC+CT	19/37	1		1	
		TT	4/16	0.3 (0.1-1.2)	0.07	0.3 (0.1-1.6)	0.17
	FLT	CC+CT	7/23	1		1	
		TT	16/30	2.6 (0.8-8.2)	0.08	1.9 (0.5-7.8)	0.36
	PDGFRα	GT+TT	4/19	1		1	
		GG	19/34	4.8 (1.3-17.3)	0.01	6.6 (1.4-31.4)	0.02
Hypertension	Gender	male vs	22/40	1			
		female	5/13	0.2 (0.1-0.9)	0.04		
	CYP3A5	AA+AG	17/24			1	
		GG	13/29	0.3 (0.1-1.1)	0.05	0.3 (0.1-0.9)	0.05
	ABCB1	other	12/16			1	
		TT+AT+GT	18/37	0.3 (0.08-1.2)	0.07	0.8 (0.2-3.9)	0.82
	ABCG2	CC+AC	24/43	1		1	
		AA	6/10	1.2 (0.3-4.8)	0.55	4.4 (0.7-26.5)	0.10
	ABCB1’	CC+CT	19/26	1		1	
		TT	11/27	0.3 (0.1-0.8)	0.02	0.4 (0.1-1.4)	0.14
	ABCB1’’	CC+CT	21/37	1		1	
		TT	6/16	0.5 (0.1-1.5)	0.16	0.5 (0.1-2.0)	0.29
	FLT	CC+CT	9/23	1		1	
		TT	18/30	2.3 (0.8-7.1)	0.11	2.9 (0.7-12.2)	0.14
	PDGFRα	GT+TT	9/19	1		1	
		GG	21/34	1.7 (0.6-5.6)	0.23	1.1 (0.3-4.6)	0.89
Thrombocytopenia	Gender	male vs	21/40	1			
		female	5/13	0.6(0.2-2.0)	0.29		
	CYP3A5	AA+AG	12/24	1		1	
		GG	14/29	0.9 (0.3-2.8)	0.56	1.2 (0.3-4.4)	0.77
	ABCB1	other	9/16	1		1	
		TT+AT+GT	17/37	0.7 (0.2-2.2)	0.35	1.7 (0.4-8.1)	0.48
	ABCG2	CC+AC	20/43	1		1	
		AA	6/10	1.7 (0.4-7.0)	0.34	2.3 (0.4-12.0)	0.34
	ABCB1’	CC+CT	13/26	1		1	
		TT	13/27	3.1 (0.8-12.7)	0.07	0.6 (0.2-2.5)	0.50
	ABCB1’’	CC+CT	19/37	1		1	
		TT	7/16	0.7 (0.2-2.4)	0.42	1.0 (0.2-4.0)	0.97
	FLT	CC+CT	9/23	1		1	
		TT	17/30	2.0 (0.7-6.1)	0.16	2.2 (0.6-8.2)	0.25
	PDGFRα	GT+TT	5/19	1		1	
		GG	21/34	4.2 (1.2-14.5)	0.02	5.2 (1.3-21.8)	0.02
Diarrhea	Gender	male vs	19/40	1			
		female	5/13	1.4(0.4-5.2)	0.40		
	CYP3A5	AA+AG	13/24	1		1	
		GG	11/29	1.9(0.6-5.8)	0.18	2.0(0.6-7.3)	0.29
	ABCB1	other	8/16	1		1	
		TT+AT+GT	16/37	1.3(0.4-4.3)	0.44	1.6(0.4-7.1)	0.55
	ABCG2	CC+AC	18/43	1		1	
		AA	6/10	0.5(0.1-2.0)	0.25	0.5(0.1-2.5)	0.38
	ABCB1’	CC+CT	14/26	1		1	
		TT	10/27	2.0(0.7-5.9)	0.17	1.6(0.4-6.2)	0.47
	ABCB1’’	CC+CT	16/37	1		1	
		TT	8/16	0.8(0.2-2.5)	0.44	1.3(0.3-5.3)	0.73
	FLT	CC+CT	14/23	1		1	
		TT	10/30	3.1(1.0-9.6)	0.04	3.2(0.9-11.7)	0.08
	PDGFRα	GT+TT	9/19	1		1	
		GG	15/34	1.1(0.4-3.5)	0.52	1.3(0.3-5.2)	0.68
Leucopenia	Gender	male vs	12/40	1			
		female	2/13	2.4(0.5-12.3)	0.26		
	CYP3A5	AA+AG	5/24	1		1	
		GG	9/29	0.6(0.2-2.1)	0.30	0.5(0.1-2.1)	0.36
	ABCB1	other	3/16	1		1	
		TT+AT+GT	11/37	0.5(0.1-2.3)	0.32	0.2(0.0-1.5)	0.12
	ABCG2	CC+AC	10/43	1		1	
		AA	4/10	0.5(0.1-1.9)	0.24	0.5(0.1-2.8)	0.45
	ABCB1’	CC+CT	6/26	1		1	
		TT	8/27	0.7(0.2-2.4)	0.41	1.6(0.3-7.2)	0.56
	ABCB1’’	CC+CT	9/37	1		1	
		TT	5/16	0.7(0.2-2.6)	0.42	0.8(0.2-3.7)	0.78
	FLT	CC+CT	7/23	1		1	
		TT	7/30	1.4(0.4-4.9)	0.39	1.2(0.3-5.2)	0.79
	PDGFRα	GT+TT	4/19	1		1	
		GG	10/34	0.6(0.2-2.4)	0.37	0.4(0.1-1.7)	0.19
Dose reduction	Gender	male vs	13/40	1			
		female	6/13	1.8(0.5-6.4)	0.29		
	CYP3A5	AA+AG	9/24	1		1	
		GG	11/29	1.0(0.3-3.1)	0.60	1.0(0.2-3.8)	0.95
	ABCB1	other	10/16	1		1	
		TT+AT+GT	10/37	0.2(0.1-0.8)	0.02	0.2(0.0-1.2)	0.08
	ABCG2	CC+AC	16/43	1		1	
		AA	4/10	1.1(0.3-4.6)	0.57	0.8(0.2-4.3)	0.84
	ABCB1’	CC+CT	9/26	1		1	
		TT	10/27	1.1(0.4-3.4)	0.54	2.1(0.5-9.0)	0.34
	ABCB1’’	CC+CT	14/37	1		1	
		TT	5/16	0.7(0.2-2.6)	0.45	0.5(0.1-2.3)	0.39
	FLT	CC+CT	11/23	1		1	
		TT	8/30	0.4(0.1-1.3)	0.10	0.2(0.1-0.9)	0.04
	PDGFRα	GT+TT	5/19	1		1	
		GG	14/34	2.0(0.6-6.7)	0.22	1.7(0.4-7.2)	0.49

### The effect of SNPs present in cell-free DNA on the population PK of sunitinib in RCC patients

In order to maximize the clinical benefits of sunitinib, an effective therapeutic management strategy with dose optimization is essential. The objective of this analysis was to describe the PK of sunitinib by a population PK approach using the collected 127 PK samples and to quantitatively evaluate the effect of potential predictive factors, including *ABCB1* genotype, on the PK of sunitinib. A one-compartment model for sunitinib was structured as schematically shown in Supplementary Figure 1. The base PK model was designed based on objective function values. The final model for the sunitinib molecule was a one-compartment model with first-order adsorption. CL/F and Vd/F were estimated to be 21719 mL/h and 112753 mL. −2LL was 982. No covariates (BW, age, sex and SNPs) had a relationship with Vd/F and CL/F parameters. Sunitinib and SU12662 were modeled simultaneously. BW and the ABCB1 rs2032582 (Z_3_) genotypes of SU12662 had a remarkable effect on apparent clearance of SU12662. Sex, age and other genotypes did not affect sunitinib pharmacokinetics. Values of the parameter estimates for the base model and final model of sunitinib are shown in Table 6. The final regression model is shown in the following equation (final equation):

**Table 6:** Values of parameter estimates of sunitinib

Parameter	Estimate	CV%	Shrinkage
**Base model**			
tvKa (h-1)	0.0119567	10.9	--
tvV/F (mL)	112753	20.7	--
tvCl/F (mL/h)	21719	4.75	--
tvCMultStdev	0.324902	7.89	--
stdev0	7.40642	22.90	--
**Final model**			
tvKa (h-1)	0.0117	11.4	0.386
tvV (mL)	99438	43.4	0.773
tvV2 (mL)	916641	50.7	0.975
tvClp (mL/h)	24576	6.21	0.317
tvClm (mL/h)	53614	9.9	0.163
tvCMultStdev	0.31	13	
tvC2MultStdev	0.242	14.5	
dClmdBW	0.538	63.7	
dClmdZ31	0.314	60.5	
dClmdZ32	0.269	68.4	
dClmdZ33	0.308	62.1	
dClmdZ34	0.0368	19.8	
dClmdZ35	0.0456	7.58	
stdev0	0.0751	12.9	
stdev1	1.23	37.5	

CLm/F = 53614 × (weight (kg)/68.3)^0.538^ × (1 – 0.314 × (Z_3_ = 1)) × (1 – 0.269 × (Z_3_ = 2) × (1 – 0.308 × (Z_3_ = 3)) × (1 – 0.0368 × (Z_3_ = 4)) × (1 – 0.0456 × (Z_3_ = 5))

When Z_3_ = 0 for AT, 1 for TG, 2 for GG, 3 for TT, 4 for AG, and 5 for GT

(final equation)

CLm/F was represent for the the apparent volume of the central compartment cleared of drug per unit time was estimated using the formula (and m represent for the metabolite SU12662). Z3 was represent for ABCB1 rs2032582.

**Figure 2:**
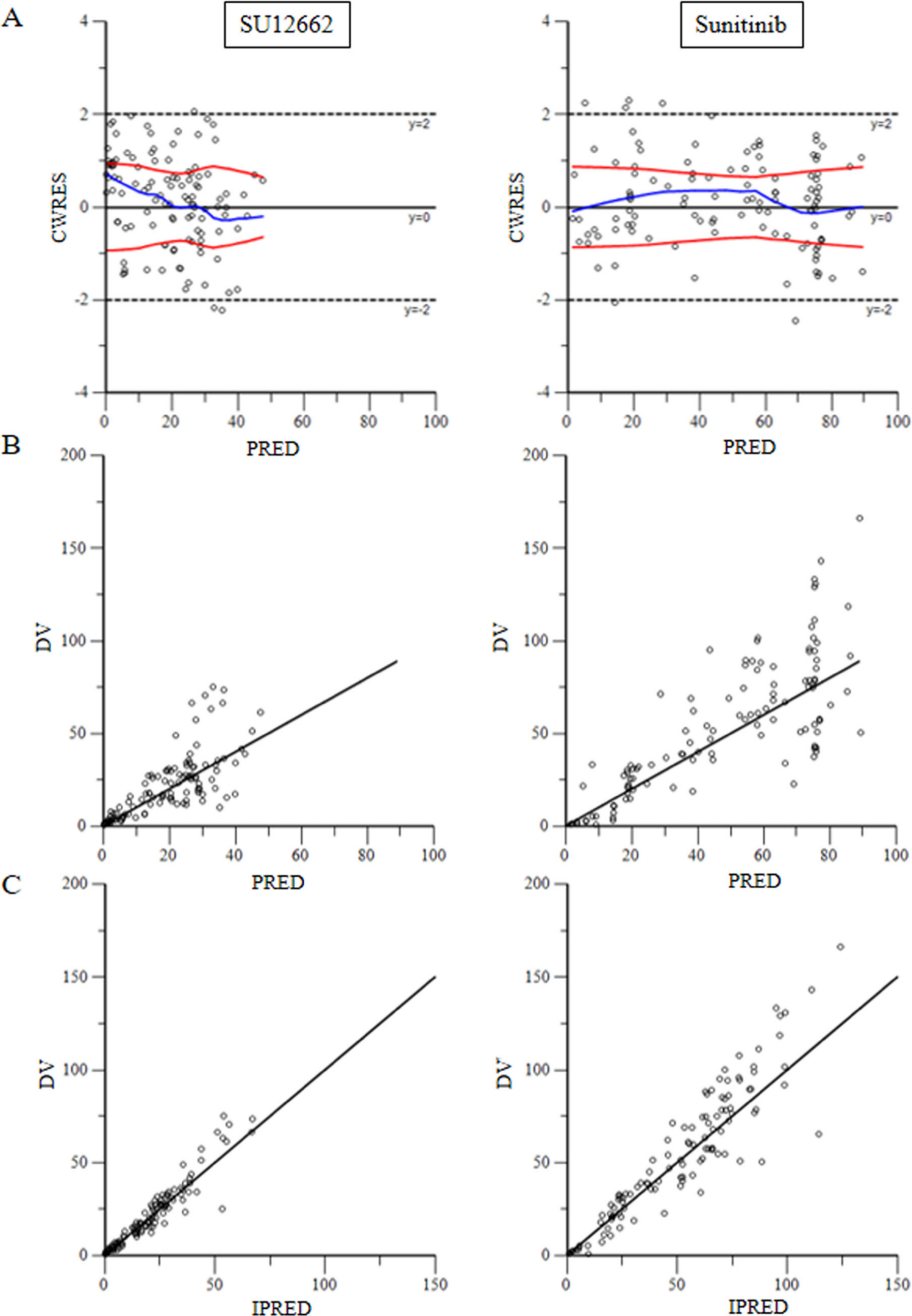
(**A**) for plots of predicted variable in the x-axis and CWRES (Conditional Weighted Residuals) in the y-axis. (**B**) for plots of dependent variable in the x-axis and predicted variable in the y-axis. (**C**) for scatter plot of the dependent variable (DV, concentrations for PK models) versus individual predicted values (IPRED, predicted concentrations).

To test the goodness-of-fit of the final model, plots were generated including CWRES versus PRED, DV versus PRED and DV versus IPRED (Figure 2). These plots show the goodness of fit of the final model. Bootstrap analysis results are shown in Table 7. And the VPC results are shown in Supplementary Figure 1 and Supplementary Figure 2. The population pharmacokinetic parameter estimates of the final models were similar in the median of the parameters estimated during the bootstrap process and the 95% confidence intervals largely overlapped for the pharmacokinetic parameters of the sunitinib models.

**Table 7:** Bootstrap results

Parameter	Estimate	CV%	BootStrap Median	BootStrap 95% CI
tvKa (h^−1^)	0.0117	11.4	0.0117	[0.00892, 0.0140]
tvV (mL)	99438	43.4	101263	[4459, 191880]
tvV2 (mL)	916641	50.7	928793	[415037, 1569500]
tvClp (mL/h)	24576	6.21	24175	[21769, 27036]
tvClm (mL/h)	53614	9.9	53331	[39962, 64261]
tvCMultStdev	0.31	13	0.277	[0.169, 0.338]
tvC2MultStdev	0.242	14.5	0.24	[0.194, 0.315]
dClmdBW	0.538	63.7	0.658	[0.00691, 1.41]
dClmdZ31	0.314	60.5	0.301	[0.00247, 0.929
dClmdZ32	0.269	68.4	0.233	[0.00200, 0.927]
dClmdZ33	0.308	62.1	0.318	[0.000875, 0.891
dClmdZ34	0.0368	19.8	0.0537	[0.00581, 0.860]
dClmdZ35	0.0456	7.58	0.0502	[0.0267, 0.320]
stdev0	0.0751	12.9	0.127	[0.0000526, 6.33]
stdev1	1.23	37.5	1.09	[0.00464, 1.61]

## DISCUSSION

We analyzed the association between sunitinib-induced AEs, SNPs, and PK in Chinese RCC patients treated with sunitinib. *CYP3A4* and *CYP3A5* are the main enzymes involved in the metabolism of sunitinib [9, 23], *ABCB1* and *ABCG2* are believed considered to participate in the absorption of sunitinib [24], and *PDGFR* and *FLT* are the two of the most important targets of sunitinib [23, 25]. Gene polymorphisms in these enzymes may have a significant impact on the efficacy and adverse reactions of sunitinib.

In our study, we found that 45% of Chinese RCC patients underwent dose reduction because of AEs of sunitinib. The percent of dose reduction was higer than the Caucasian population [26–29], and lower the Japanese and Koreans population [6, 24, 28]. When compared to a multicenter study research data [30, 31], the frequency of HFS, hypertension and hypothyroidism in Chinese RCC patients is clearly higher than in other countries. Our study also found that patients with grade 3 AEs had a higher plasma level of sunitinib or SU12662 than patients without grade 3 AEs. but these findings suggest an exposure-toxicity relationship of sunitinib. Interestingly, this finding was consistent with a prospective study that conducted among Australia patients [32].

Although the relationship between SNP and AE of sunitinib has been reported in other studies, there are few research about Chinese sunitinib-treated patients. In our study, we found that a higher severity of AEs were collected with SNP rs2032582 in ABCB1. ABCB1 genotype as a predictive covariate for apparent oral clearance of sunitinib. These results are consistent with previously reported data [13, 33–35]. However, we did not find the variant ABCG2 421C > A is suggestively associated with severe thrombocytopenia, this was different with Low SK finding [36]. More interestingly, we firstly demonstrated that necessary dose reductions of sunitinib were significantly correlated with SNP rs1933437 in FLT. Thrombocytopenia was collected with rs1800812 in PDGFRα. A higher severity of AEs (grade ≥ 2 hand-foot syndrome) was collected with rs1800812 in PDGFRα.

A population approach was used to assess the PK of sunitinib and its active metabolite SU12662, and to identify covariates that might explain variability in exposure following oral administration. The model was different with other literature [26]. This might because of the sparse time-point samples. Therefore, if possible, more plasma samples from each patients should be collected before and after oral administration, especially after administration. and more studies need to be conducted.

Many researchers have reported on the relationship between SNPs and AEs of sunitinib [3, 27, 37, 38], and many researchers have reported on the association between PK and AEs of sunitinib [39–42]. However, very few studies about the relationship between SNPs, PK and AEs have been reported for Chinese RCC patients treated with sunitinib.

The population pharmacokinetic model we established, was to quantitate the covariate influence on pharmacokinetic parameters. Due to the small sample size, the result could not service for clinical directly, but could provide a scientific basis for individualized treatment, and provide a methodology reference for the similar drugs.

## CONCLUSIONS

Our study preliminarily confirmed the hypothesis that the PK of sunitinib is affected by enzyme polymorphisms in Chinese RCC patients treated with sunitinib, and this affects the different distribution and severity of AEs of sunitinib.

## MATERIALS AND METHODS

### Study population and clinical collection

We consecutively enrolled histologically confirmed RCC patients who were treated with sunitinib and were available for PK analysis and genetic analysis from March 2014 at Academy of Military Medical Sciences Affiliated Hospital, Beijing, China. Demographic and clinical data of patients were collected from the review of electronic medical records. Patient characteristics considered relevant for sunitinib toxicity were as follows age, sex, BSA, Eastern Cooperative Oncology Group (ECOG) performance status, histologic type, and organ function.

### Study design

Sunitinib was administered orally as a single agent at a dosage of 50 mg daily for 4 weeks followed by a 2-week off period (schedule 4/2) until progression or intolerable toxicity occurred. Dose reductions of sunitinib were allowed depending on the type and severity of AEs. All adverse effects were graded by the attending doctors according to National Cancer Institute-Common Terminology Criteria for Adverse Events v. 3.0 (CTCAE 3.0).

On the basis of clinical relevance and grading objectiveness, we analyzed sunitinib treatment-emergent AEs, including HFS, hypertension, diarrhea, fatigue, a loss of appetite, leukopenia, and thrombocytopenia. Data on dose reductions were documented for the first four cycles of sunitinib treatment. We also recorded adverse toxic events leading to dose reductions and the date on which they occurred.

This study was conducted in accordance with Good Clinical Practice and under the ethical principles established by the Declaration of Helsinki. Each protocol was reviewed and approved by the Institutional Review Board of the Academy of Military Medical Sciences Affiliated Hospital, and all patients gave written informed consent.

### Sunitinib pharmacokinetic analysis

Samples for PK analysis were collected at day 15 ± 1 after sunitinib treatment. Blood samples were drawn into 2 mL EDTA vacutainer tubes and, thereafter, directly sent to the laboratory, after centrifugation at 1500 *xg* for 5 minutes at 4°C, plasma was transferred to propylene tubes and stored at −70°C until assay of plasma. Sunitinib concentrations in plasma were determined using a validated method. Supplementary Table 2 summarizes the intra and inter-day precision and accuracy values for the QC samples. The intra- and inter-day precisions for sunitinib were < 1.64%, while accuracy was within ±5.69%. The intra- and inter-day precisions for SU12662 were < 12.4%, while accuracy was within ±12.74%. The accuracy and precision data indicate that method is reliable and reproducible.

### DNA isolation and analysis of polymorphisms

Free-circulating DNA was isolated from 1 mL plasma using a QIAamp Circulating Nucleic Acid Kit (Qiagen, Hilden, Germany) according to the manufacturer's protocol. Prior to analysis, DNA concentrations were measured using amicroplate reader (Biotek, Vermont, American). Genotyping was performed with an ABI 3730xl DNA Analyzer (Applied Biosystems) using a LifePro Thermal Cycler (Hangzhou Bioer Technology Co., Ltd.). The primer sequences information was shown in Supplementary Table 3.

Six polymorphisms in four genes involved in general pharmacokinetics and others involved in the pharmacodynamics of sunitinib were selected for analysis based on the functionality evidence and clinical relevance reported by previous studies [3, 25, 43]. We genotyped 8 SNPs in 6 candidate genes, *CYP3A4* (rs35599367), *CYP3A5* (rs776746), *ABCB1* (rs1045642, rs1128503 and rs2032582), *ABCG2* (rs2231142), *PDGFRα* (rs1800812), and *FLT3* (rs1933437).

### Population PK study

#### Model development

Population PK analysis was performed using Pheonix (version 1.4) with (nonlinear mixed effect modeling) NLME. First order conditional estimates (FOCE) were applied for all estimations. Interindividual variability was assessed using an exponential variability model for continuous covariates (body weight and age) (Equation 1) and a linear proportional model for categorical covariates (Equation 2).

Pi=Ppop×exp (ηi) (1)

Pi=Ppop×(1+ηi) (2)

Where *P*i represents the value of the PK parameter for the *i*th individual, Ppop is the population mean for P and η is an interindividual random effect with a mean of zero and variance of ω^2^. Residual unexplained variability was evaluated using a combined proportional and additive error model (Equation 3).

CObs=C+CEps+C*CEps*CMixRatio (3)

Where CEps represents the additive error and Ceps *CmixRatio is the proportional error. All compartment models were parameterized in terms of values of apparent oral clearance (CL/F), volume of distribution (Vd/F) and absorption rate constant (Ka). The models were assessed and selected based on goodness of fit and a variety of criteria including physiological plausibility and stability. Comparative evaluation among the covariate models was based on the −2*Log (likelihood) (−2LL). A decrease in −2LL of 3.84 (*p* < 0.05 assuming a *X*2 distribution) was considered to be significant for the forward addition and 6.63 (*p* < 0.01) for the backward elimination. The models were also compared using the Akaike information criterion (AIC = −2LL + (2 × nParm)) to discriminate between nonhierarchical models in the selection of a structural model.

Patient characteristics, including body weight, sex, age and genotype were evaluated as covariates. An exploratory analysis was used to assess the relationships between PK parameters and covariates by visually inspecting plots of the empirical Bayesian estimates. Body weight (BW) and age were evaluated as covariates by applying allometric scaling to CL/F and Vd/F as described in equation 4:

$$\text{Pi}=\text{Pop}\times {\left(\text{BW or age/the average of BW or age}\right)}^{\text{power}}$$ (4)

Where the average BW was 68.3 kg and the average age was 52. Categorical covariates, such as sex and genotype were assessed with a linear proportional model. Covariates were added into the base model using a forward stepwise inclusion approach until there was no further decrease in −2LL. Covariates were then removed from the model using a backward stepwise approach.

### Model evaluation

The following diagnostic plots were used to evaluate the models: dependent variable (observed value) (DV) versus population predicted value (PRED), DV versus individual predicted value, conditional weighted residuals (CWRES) versus PRED, and CWRES versus time after the dose, to identify bias corresponding to model miss-specification. η-shrinkage was evaluated by post hoc Bayesian estimates-based diagnostic method. The final model was evaluated using nonparametric bootstrap analysis and visual predictive check (VPC). A thousand bootstrap runs were generated by random resampling using the original data set. Standard errors of population parameter estimates and random effects error models were also evaluated.

### Statistical analysis

Two independent samples *t* test was used to compare the steady state concentration of each dose group between the different severity of adverse reaction, when *p* < 0.05, indicating a significant difference. Genotype frequencies at each locus were tested for Hardy-Weinberg equilibrium using a χ^2^ test. For toxicity analysis, baseline corrected toxicity scores were calculated by subtracting baseline values from the maximum recorded score in four cycles of treatment, and the HFS toxicity end point was dichotomised as higher than grade 1 (yes or no). The other toxicity end point was dichotomised as happened versus unhappened. The end-point dose reduction was dichotomised as any dose reduction within cycle 1-4 or no dose reduction. Genotype associations with toxicity events were first analyzed using univariate and multivariate logistic regression. For multivariate analyses, associations with *p* ≤ 0.05 were considered significant. Statistical analyses were performed using SPSS19.0 software (IBM Corp., Armonk, NY, USA).

All the result of plasma concentrations of sunitinib and the active metabolite and the patient characteristic that corrected from this study were used in the population PK study, and weight, sex, age and genotypes were included as covariates. Population pharmacokinetics study was performed using Phoenix software (Version1.4, Pharsight, A Certara Company, USA).

## SUPPLEMENTARY MATERIALS FIGURES AND TABLES



## Abbreviations

SNP: single-nucleotide polymorphisms
SU12662: N-desethyl sunitinib
mRCC: metastatic renal cell carcinoma
HFS: hand-foot syndrome
VEGFRs: vascular endothelial growth factor receptors
PDGFR: platelet-derived growth factor receptor
FLT3: Fms-like tyrosine kinase 3 receptor
RET: receptor encoded by the ret proto-oncogene
CYP1A1: pharmacokinetic related genes including cytochrome P450 1A1
ABCG2: ATP binding cassette member G2
ABCB1: ATP binding cassette member B1
cfDNA: cell free DNA
AEs: adverse events
